# Fetal ECG Extraction from Abdominal Signals: A Review on Suppression of Fundamental Power Line Interference Component and Its Harmonics

**DOI:** 10.1155/2014/239060

**Published:** 2014-02-09

**Authors:** Dragoş-Daniel Ţarălungă, Georgeta-Mihaela Ungureanu, Ilinca Gussi, Rodica Strungaru, Werner Wolf

**Affiliations:** ^1^Applied Electronics and Information Engineering Department, Politehnica University of Bucharest, 061071 Bucharest, Romania; ^2^University of Medicine and Pharmacy Carol Davila, 050474 Bucharest, Romania; ^3^Institut für Infomationstechnik, Universität der Bundeswehr München, 85577 Neubiberg, Germany

## Abstract

Interference of power line (PLI) (fundamental frequency and its harmonics) is usually present in biopotential measurements. Despite all countermeasures, the PLI still corrupts physiological signals, for example, electromyograms (EMG), electroencephalograms (EEG), and electrocardiograms (ECG). When analyzing the fetal ECG (fECG) recorded on the maternal abdomen, the PLI represents a particular strong noise component, being sometimes 10 times greater than the fECG signal, and thus impairing the extraction of any useful information regarding the fetal health state. Many signal processing methods for cancelling the PLI from biopotentials are available in the literature. In this review study, six different principles are analyzed and discussed, and their performance is evaluated on simulated data (three different scenarios), based on five quantitative performance indices.

## 1. Introduction

The fetal heart rate (fHR) and the morphological analysis of the fetal electrocardiogram (fECG) are two of the most important tools used nowadays in clinical investigations to examine the health state of the fetus during pregnancy. The fHR is the mostly used parameter in fetal monitoring, since 1818 [1]. While the fHR track shows a predictive value of almost 99% for the fetal well being investigation, an abnormal fHR has a predictive value of only 50%. Hence, it provides relatively poor specificity in detecting the fetal distress [2]. Additional information about the fetal well being can be obtained by analyzing the morphology of the fECG signal, which was recently introduced in clinical practice for fetal monitoring. Its clinical relevance was demonstrated by a series of clinical studies [3], randomized controlled trials [4–8] and prospective observational studies [9–18], which prove that clinical fetal monitoring based on both fHR and fECG morphology analysis, especially the ST waveform analysis, leads to the reduction in the number of operative vaginal deliveries, smaller rate of metabolic acidosis at birth, less blood samples performed during labor, and fetal morbidity reduction.

The standard procedure to record the fHR is the cardiotocography (CTG), sometimes known as electronic fetal monitoring [19]. When necessary to investigate both the instantaneous fHR and the fECG morphology, an invasive fetal monitoring method that uses a wire electrode attached to the fetal scalp [20], after the membrane rupture, is preferred. However, both methods have important drawbacks: (i) the fHR obtained via CTG has the potential problems of reliability and accuracy [21, 22]; in addition, the beat-to-beat variability of fHR is not present in the CTG traces [23, 24]; hence, rapid variations of the fHR cannot be detected; (ii) the second recording technique is invasive [20]; thus, it can put the life of both the mother and the fetus in danger (e.g., possible infections can lead to different complications).

An alternative method to obtain the instantaneous fHR and the fECG morphology is the abdominal recording of the fECG which considers an array of electrodes placed on the maternal abdomen. This recording procedure overcomes the main drawbacks of the methods used in clinical routine for fetal monitoring. However, the limitation of this technique is the very low signal-to-noise-ratio (SNR) of the available recorded fECG. This is mainly due to the fact that the fECG signal is generated by a small source (fetus heart). In addition, it has to propagate through different attenuating media to reach the maternal belly surface. Hence, the fECG signals contained in abdominal signals (ADSs) provide an amplitude of about 10 *μ*V [24] which becomes still smaller around 28th until 32nd weeks of gestational age due to the appearance of the insulating layer called *vernix caseosa*.

**Figure 1:**
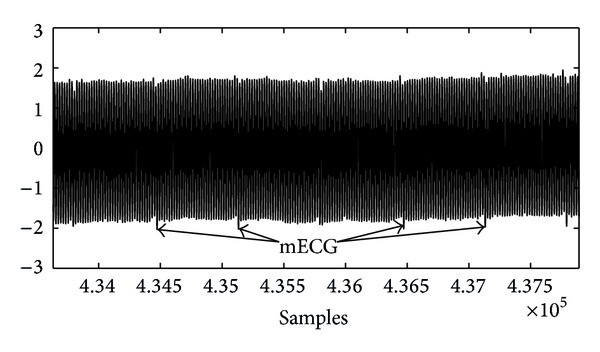
Abdominal signal affected by PLI including harmonics.

Furthermore, the signal of interest, that is, the fECG, is only one (weak) component of the ADS mixture; other (disturbing) signals with higher power that also exist are the electromyogram (EMG) of the abdominal muscles, the electrohysterogram (EHG), the maternal ECG (mECG), the baseline wander basically due to the maternal respiration, and the power line interference (PLI). Among them, the PLI, with the fundamental PLI component of 50 Hz/60 Hz, and its harmonics is one of the most disturbing noise sources, because it can reach amplitudes much greater than the abdominal fECG signal, making its analysis almost impossible (see Figure 1).

The PLI is determined by the power supply network, and its appearance in the abdominal recordings is explained by (i) the electrostatic induction and parasitic capacitance coupling between the body and the ground; and (ii) the electromagnetic induction through loops of the recording cables, where a time-varying magnetic field generates a voltage proportional to the loop area (depending on its orientation) and to the strength of the magnetic field [25]. If the cables are twisted, the induced voltage is reduced [25], but still significant for the fECG analysis.

The fundamental PLI is definitely a problem in fECG analysis, and its harmonics, usually present, make the PLI cancelling problem even more complex. The harmonics are usually generated by connected nonlinear loads: neon lamps, TVs, microwaves ovens, fridges, air conditioning devices, computers, and basically almost any power electronics device connected to a single-phase distribution system. The disturbing sources are in fact the rectifiers and semiconductor switches present in almost all of these nonlinear loads which introduce distortions in the power supply waveforms [26, 27]. Surprisingly, the 3rd harmonic, that is, 150 Hz/180 Hz, is the most powerful PLI harmonic [28].

Although there are many practical solutions to reduce the PLI, for example, the cable twisting and shielding, the use of differential recording involving an instrumentation amplifier with high common mode rejection ratio (CMRR) at power line frequency, and the proper skin preparation to reduce the electrode imbalance, the PLI still affects the biopotential measurements. For example, the advantage of high CMRR instrumentation amplifiers is limited in real life applications, since a slight imbalance in the electrode-skin impedance leads to the divider effect [25]; thus, the PLI is partly transformed into a differential signal [25, 29, 30] which passes through the amplifier together with the signal of interest, when no notch filter is available in the amplifier circuit to suppress the PLI components. However, the spectrum of the ECG signal for neonates ranges between 0.01 Hz and 250 Hz [31], while the abdominal fECG frequencies are supposed to be up to 500 Hz; thus, such a notch filter affects also the signal of interest. Finally, any PLI disturbance, even if much attenuated, impairs the morphological analysis of the abdominal fECG due to its very low amplitude.

There are many processing methods available in the literature, addressing the PLI suppression in biopotential recordings. The main PLI cancelling techniques are (i) fixed-frequency digital notch filters [32–36], (ii) adaptive filters [37–42], (iii) time-frequency processing of nonstationary signals (wavelet transform) [43–45], (iv) time-frequency nonlinear analysis of nonstationary signals [46–48], (v) Kalman filters [49, 50], (vi) neural networks [51, 52], (vii) blind source separation [53–55], (viii) spectral Hampel filter [56], and (ix) subtraction procedure [57, 58]. These nine categories can be further grouped into nonmodel techniques (a) (iii, iv, vii, and viii) and model based techniques (b) (v, vi, and ix).

From this large variety of PLI suppression methods, six representative approaches are selected in this review study: digital notch filters (DNF), adaptive filters (AF), Hilbert Huang transform (HHT), wavelet transform (WT), blind source separation (BSS), and neural networks (NN); they are briefly described in the following sections which discuss their advantages and disadvantages. A recent and representative algorithm is implemented for each PLI cancelling approach and is evaluated using simulated data. The signal processing method with the best performance in PLI reduction, considering the minimal distortion of the original signal as the evaluation criterion, is identified.

## 2. Materials and Methods

### 2.1. PLI Cancellation Using Digital Fixed Notch Filters

Notch filters are used to cancel narrow band interferences, one common application being the PLI suppression in biopotential measurements. Digital fixed notch filters can be designed to remove multiple frequencies, having the advantage of being designed to remove the fundamental frequency and also its harmonics (multiple-notch filters or comb filters [34, 59]).

However, the main problem of multiple-notch filters, when used for cancelling the PLI signal from fECG signals, is the fECG and PLI spectral overlapping. Thus, the notch filter should have a very narrow bandwidth in order to suppress mainly the 50 Hz and its harmonic components, and not the useful information contained in the fECG spectrum. But this requirement comes into conflict with the fact that actually the real PLI signal does not have a fixed fundamental frequency, but rather a frequency that varies around the value of 50 Hz which requires a wide bandwidth of the multiple-notch filter. Moreover, the transient time introduced by the notch filter can be too long, in which case the fECG can be considerably distorted. Hamilton [60] has investigated the effect of the transient time of the notch filters, which increases much when the bandwidth is decreased. They observed the ringing effect appearing near the QRS complex and ST segment when narrow bandwidth notch filters are used. This distortion decreases when the transient time increases [60, 61]. However, in practice, a long transient time reduces the capacity of the filter to track the noise level changes [60]. The latest available international standards for ECG acquisition, American National Standard Association for the Advancement of Medical Instrumentation (AAMI) [62] and International Standard IEC 60601 [63], do not specify any requirements for the transition band of the notch filters. The only specification present in the IEC 60601 standard states that “notch filters for line frequency interference suppression shall not introduce on the ECG record more than 25 *μ*V peak ringing noise” [63]. Nevertheless, the notch filter should have a short transient time, minimal distortion, and very narrow bandwidth.

Pei and Tseng [64] propose a method to decrease the transient time of multiple IIR notch filters. This technique uses the vector projection in order to find better initial values for the IIR notch filter. A more recent paper [32] reports better results in suppressing the transient time than the ones obtained when applying the method introduced in [64]. Piskorowski proposes a time-variant multiple-notch IIR filter. The transient time is reduced by varying the pole radius with time and thus the filter is able to cancel the fixed frequencies PLI components as fast as possible, with no long-term selectivity impairment [32]. This type of filter should not be confused with adaptive filters, which are varying the notch central frequency, as is explained in the next section.

*The time-varying multiple-notch IIR filter (TVMNF)* proposed in [32] is chosen as representative for this category of power line PLI suppression methods. Thus, the general transfer function of the multiple-notch filter is

(1) H(z)=∏i=1K1−2cos⁡(ΩNi)z−1+z−21−2rcos⁡(ΩNi)z−1+r2z−2=B(z)B(r−1z),B(z)=∑i=02Kbiz−i,

where *K* is the number of notches, *Ω*_*Ni*_ is the central frequency of the notch, *N* is the order of the harmonics, *B*(*z*) is a symmetrical polynomial, and *r* is the pole radius. The selectivity of the filter increases when *r* is increased, but this results also in a longer transition. Thus, the transition time is influenced by the radius *r* and in order to improve the time domain filter response, the *r* is varying in time. The difference equation of the IIR multiple-notch filter with a time-varying parameter *r* is

(2) y(n)=b0x(n)+b1x(n−1)+⋯+b2Kx(n−2K)−r(n)b1y(n−1)−⋯−r2K(n)b2Ky(n−2K),

where the variation of the pole radius varies is described by [65]

(3) $$\begin{array}{c} r\left(n\right)=\overset{-}{r}\left(1+\left(d_{r}-1\right)e^{-n/vf_{s}}\right),\;n\geq 0, \end{array}$$

with variation range $d_{r}=r(0)/\overset{-}{r}$ and $\overset{-}{r}=\lim_{n\to \infty }r(n)$; *v* includes the exponential variation of *r*(*n*) in (3), and *f*_*s*_ is the sampling frequency [32]. The value chosen for *r* is critical; a very high value generates narrow notches, but their transition time is increased, while a small *r* value leads to a decreased transition time, but less selective notches are obtained, which results in filtering out important fECG frequency components. Thus, *r*(*n*) has an exponential variation, from an initial value, *r*(0), to the desired one, $\overset{-}{r}$ [32].

**Figure 2:**
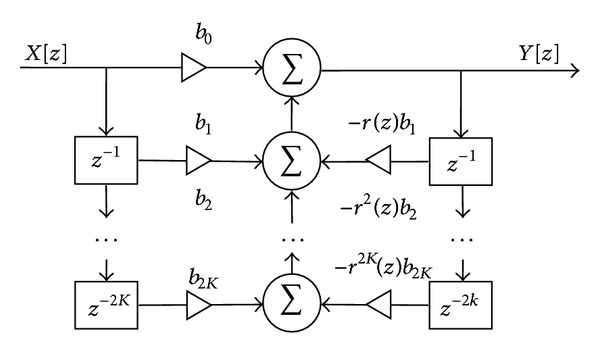
The block diagram of the TVMNF.

The filter is implemented with one notch frequency and three notch frequencies, respectively, choosing *d*_*r*_ = 0.9 and *v* = 2, as suggested in [32]. In Figure 2 a block diagram is used to describe the TVMNF algorithm.

### 2.2. Adaptive Filtering in PLI Cancellation

As previously described, fixed notch filters have the main drawback that the central frequency of the notch cannot be modified. This makes the PLI cancellation difficult when the PLI fundamental frequency has slight variations, which is often the case in real applications. To overcome this problem adaptive filters are introduced, which have the ability to adapt their notch frequency, tracking the changes in the PLI fundamental frequency. The first adaptive filter was introduced by Widrow et al., [41] and according to the review [60], this type of filters introduces less distortion than the fixed notch filters, having also a shorter transition time. The adaptive noise cancellers assume simultaneous recording of the noise source by an additional channel, that is, the reference signal. The reference is supposed to be uncorrelated with the signal of interest, but correlated with the disturbing signal. The filter adapts its parameter in order to make the reference signal as close as possible to the noise that disturbs the signal of interest, by minimizing the output error, considering the least mean square (LMS) criterion.

Many adaptive filters that suppress the PLI in ECG recordings are available in the literature. Wan et al. [67] propose a LMS adaptive algorithm with variable step size and suggest that faster convergence rate and smaller mean square error are obtained, as compared to the traditional approach. Costa and Tavares [68] come with an improvement of the basic adaptive canceller by providing also harmonics' suppression, with a minimal increase in computational complexity. Thus, the algorithm is suitable for low cost acquisition systems. Liangling et al. [42] exploit the capability of the adaptive filters to cancel the PLI component and baseline wander from ECG tracings [67].

However, these adaptive filters have a practical drawback. They all need an additional recording of the disturbing signal, that is, the reference signal. That is not always possible (e.g., in abdominal fECG recording using portable devices). Thus, adaptive filters with no reference signal are more suitable for practical applications. Ziarani and Konrad [69] propose a filter which is able to estimate the amplitude, the phase, and the frequency of the PLI components. An improved version of these adaptive filters is presented by Martens et al. [39] (Improved Adaptive Canceller—IAC), who developed an algorithm able to suppress both the PLI fundamental frequency and its harmonics. The algorithm considers the fact that large QRS amplitudes can distort the estimation of the PLI components and produce large transient segments and thus the adaptive process is blocked in such situations. The algorithm is compared with the classical adaptive filter and two notch filters with large and narrow bandwidth, respectively. The algorithm proposed by Martens et al. outperforms the other techniques, showing a stable behavior even in the worst conditions. This algorithm is chosen as representative for the adaptive filtering PLI cancellation approach and is implemented in the current study.

**Figure 3:**
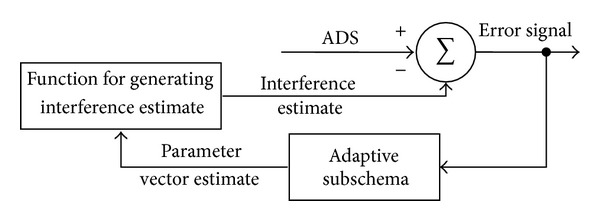
The block diagram of the general adaptive interference canceller [40].

In Figure 3 a general scheme of the adaptive notch filter is presented.

### 2.3. Blind Source Separation Applied in PLI Suppression

This PLI cancelling method is based on a completely different concept than digital filters, considering the statistical properties of a mixture of signals. Each signal source is extracted from the mixture, as long as they satisfy some conditions.

Many approaches to estimate the ICA parameters exist: maximization of nongaussianity [65, 70], maximum likelihood estimation [71, 72], tensorial methods [73], and so forth. Different research groups use ICA algorithms to extract the fECG from abdominal recorded signals: Zarzoso and Nandi 2001 [74], Vrins et al. [75], Sameni et al. [76, 77], Lathauwer et al. [73], Camargo-Olivares et al. [78], Cardoso [79], and so forth. All ICA studies report that the used ICA methods perform reasonably well in extracting the fECG signals from ADS, that is, separating the abdominal fECG from other types of noise signals, including the PLI component, present in the ADSs. It should be noticed that the interpretation of the abdominal fECG obtained via BSS methods does not have a clear physical explanation, since ICA does not take into account the position of the electrodes and other physical parameters.

**Figure 4:**
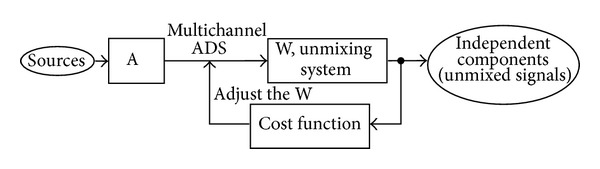
The general block diagram of the ICA algorithm.

The algorithm proposed in [65], FastICA, is chosen as representative for this PLI cancelling approach and is considered in the current study. In Figure 4 the general block diagram of the ICA concept is depicted.

### 2.4. Hilbert Huang Transform Applied in PLI Reduction

The Hilbert Huang Transform (HHT) is a powerful method for analyzing nonlinear and nonstationary time series and it was introduced by Huang et al. [80]. The method overcomes the shortcomings of the Fourier transform which is valid just for stationary time series. It is proven that Fourier transform offers a wrong energy-frequency distribution with no physical meaning when applied on nonstationary time series [80]. Taking into account that most real signals, and especially the biopotentials, are nonstationary, the HHT is suitable for their analysis.

The method has two steps: (i) generation of Intrinsic Mode Functions (IMFs) through Empirical Mode Decomposition (EMD); (ii) Hilbert analysis [80]. IMFs are fully generated from the data set and must satisfy two conditions: (a) the number of zero crossing and the number of extrema have to be equal or to differ at maximum by one; (b) the mean value of the envelope including the local maxima and the envelope defined by the local minima is zero at any point.

The generation of IMFs is fully data driven and is obtained by decomposing the time series (the process is called “sifting”) using the EMD; high frequency components are decomposed into the first IMFs, while the low frequency components are found in the higher order IMFs.

This method is reported to cancel the PLI signal from the ECG [81–83]. The basic idea is to discard the IMFs which contain noise and to reconstruct the signal from the remaining IMFs. The main problem is to identify which IMFs contain just the PLI signal. In conventional EMD the first IMF is considered to be related to the PLI signal since it contains the higher frequency and is therefore discarded, which is not necessarily true, since the sifting process is not perfect, allowing high frequency ECG components in the first IMF. Moreover, if the PLI contains harmonics, then the number of IMFs containing PLI components is higher, since the harmonics are decomposed into different IMFs.

**Figure 5:**
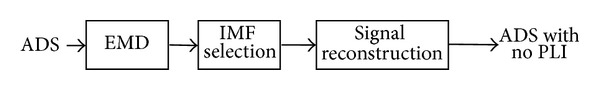
The block diagram of the HHT algorithm.

Pal and Mitra [66] propose an algorithm that identifies the IMFs containing the PLI components. It computes the IMFs' cumulative mean and their powers, and using these parameters identifies which IMFs contain PLI components, on a threshold basis. However, the algorithm is not robust when the power of the PLI signal is high as compared to the signal of interest, the fECG. Therefore, the current study improves the original HHT based PLI cancelling method, by a more precise identification of the noise IMFs being obtained. The Hilbert transform is computed for each IMF and the instantaneous frequency is derived thereafter. The IMFs containing the PLI components are then discarded; as already mentioned, these IMFs can still contain information about the high frequency ECG components, that is, the QRS complex. In order to recover the QRS complex, the IMFs with QRS complex are firstly detected, based on the instantaneous frequency, and then the algorithm described by Kabir and Shahnaz [47] is applied as follows:

- the QRS complex boundaries are identified:
  (a.1) the *R* peak locations are detected;
  (a.2) two nearest local minima, located on both sides of *R* peak are found;
  (a.3) one zero-crossing point on the left-hand side of its left minimum and the other one on the right-hand side of the right minimum are detected. The boundaries of the QRS complex are assumed to be between these two points;
- a Tukey window centered on the *R* peaks, which spans to cover the QRS complex, is applied, that is, multiplies the selected IMF. This window offers a flat gain at the *R* wave and decreases gradually to zero ensuring a smooth transition with minimal distortion. Thus, the information of the QRS complex is preserved, allowing the estimation of the fECG. In Figure 5 the block diagram of the algorithm is depicted.

### 2.5. PLI Cancellation by Applying Neural Networks

**Figure 6:**
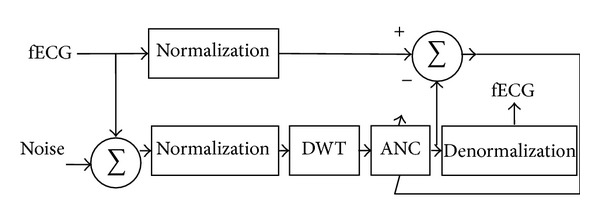
The block diagram of the WNN algorithm.

The classical application of NN in cardiac signals processing is the classification of ECG signals, pattern recognition [84, 85], and fECG extraction from ADS [86]. Methods for fECG SNR improvement are described in [87] where a Functional Link Artificial Neural Network (FLANN) is proposed to remove the Gaussian and baseline wander noise. Zhang and Benveniste [88] and Poungponsri and Yu [89] use NN combined with Wavelet transform for better results. However, in a recent article Poungponsri and Yu [51] come with an improvement of the method in [89] and the algorithm is tested also on PLI cancellation (Wavelet Neural Network—WNN). The NN based adaptive filtering approach proposed in [51] for ECG signal noise reduction removes the PLI signal by applying firstly the wavelet decomposition. The wavelet coefficients are further applied to a neural network trained to reconstruct the denoised ECG (see Figure 6). The algorithm was initially developed by the authors to cancel all the noise sources overlapping the ECG signal recorded at 360 Hz (the PLI fundamental frequency is 60 Hz).

The algorithm presented by Suranai et al. is chosen as representative for this category of methods and its steps are as follows:

(a) A real signal, not affected by the PLI, is applied to a feed-forward NN with 64 inputs, 2 hidden layers, and 1 output corresponding to the denoised ECG signal. The inputs of the NN are obtained by applying the Wavelet Packet Decomposition (WPD) using the Debauchies 4 wavelet; the thresholding (soft threshold) is then applied to reduce the high frequency noise. The resulting 64 wavelet coefficients are the inputs of the NN having 56 hidden neurons on the first hidden layer and 12 neurons on the second one. The hyperbolic tangent activation function is used for all the neurons:
  (4) $$\begin{array}{c} f\left(x\right)=\frac{1-e^{-x}}{1+e^{x}}. \end{array}$$
 The first 3350 samples of the “clean” selected signal resampled at 300 Hz (record 220 from the Massachusetts Institute of Technology (MIT-BIH) database [90]) are used for this training phase involving the back propagation algorithm (4000, for the raw signal, recorded at 360 Hz). In order to allow the NN to remove the PLI from signals recorded with different amplifier gains, the signals are initially normalized as follows:
  (5) $$\begin{array}{c} s_{\mathrm{norm}}\left(t\right)=\frac{2}{s_{\max}-s_{\min}}\cdot s\left(t\right)-\frac{s_{\max}+s_{\min}}{s_{\max}-s_{\min}}, \end{array}$$
 where *s*_max⁡_ and *s*_min⁡_ are the maximum, and, respectively, the minimum values of the signal.
 The training is then continued for the same segment, affected by the PLI, so that the NN learn to cancel the PLI noise.
(b) The noisy signals are then applied to the network (i.e., their wavelet coefficients, after thresholding); the denoised signal is considered as the output of the network.

It should be noticed that the algorithm presented above is adapted in this study to remove the PLI signal with the fundamental frequency of 50 Hz and for a sampling frequency of 1000 Hz.

### 2.6. PLI Reduction by Applying the Wavelet Transform

In the recent years, discrete wavelet transforms and thresholding techniques have been used for ECG denoising [91]. Wavelet based noise cancelling techniques became very popular because they are able to decompose the signal into time-frequency domain which is appropriate for the analysis of nonstationary signals. It is reported in the literature that discrete wavelet transform does not introduce any artificial information to the original signals; the threshold is generated based on the attributes extracted from the signal [92, 93].

**Figure 7:**
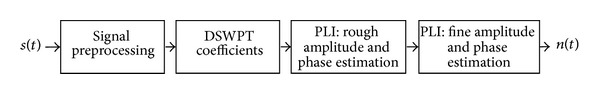
The block diagram of the DSWPT algorithm. *d*(*t*) is the signal of interest contaminated with the PLI signal, and *n*(*t*) represents the estimation of the PLI signal.

The main problem is the identification of the mother wavelet, the level of decomposition, and the optimal threshold. Garg et al. [94] compare different mother wavelet functions for ECG denoising and conclude that the recovery of the ECG with minimal artifacts is obtained when using Sym10 decomposition at level 5 and hard shrinkage function with either rigorous SURE or heuristic SURE threshold [94]. More recently, Galiana-Merino et al. [43] use the discrete stationary wavelet packet transform (DSWPT) to suppress the PLI signal and its harmonics from electromyographic (EMG) signals (the DSWPT algorithm). Basically it is a shift invariant transform to isolate the 50 Hz and its harmonics, with the disturbing sine signals being reconstructed thereafter using the DSWPT coefficients. In Figure 7 the steps of the DSWPT algorithm are described as follows.

- The linear trend is removed from the signal.
- DSWPT is applied to the detrended signal using the Meyer wavelet and considering the maximum decomposition level fixed to 3, to allow the identification of 50 Hz and of its harmonics.
- The amplitude and the phase of the sine noise signals are roughly estimated by dividing the DSWPT coefficients associated with the disturbing frequencies into segments of 20 samples which are then averaged to obtain templates for the sine wave disturbances. Based on this template some pure sine signals are generated having the amplitudes equal to the maximum values of the sine templates. The correlation between these pure sine signals and the corresponding templates allow the roughly determination of the phase shift of the PLI.
- The amplitude and the phase of the sine disturbances are further adjusted, for a better estimation of the PLI. Firstly, the best phase shift is computed by varying the phase shifts in the range [−10, 10] samples around the roughly estimated phase shifts. Secondly, the computation of the correlation between the shifted pure sine and the signal is performed. Then, the amplitudes are refined by analyzing the correlation between the signal and the pure sine waves with the amplitudes varying in the range [0.6, 1.4] around the roughly estimated amplitudes. The variation step is equal to 0.01% of the roughly estimated amplitude.
- The refined sine disturbances are subtracted from the signal that has to be denoised.

The main results regarding the performance of the selected algorithms are summarized in Table 1.

### 2.7. Data Simulation and Performance Measurements

The simulated data, used to quantitatively estimate the performance of the proposed algorithms are generated in two steps. Firstly, the fECG is simulated using the dynamic model introduced in [95, 96]:

(6) $$\begin{array}{c} \dot{x}=\alpha x-\omega y,\\ \dot{y}=\alpha y-\omega x,\\ \dot{z}=-\underset{i}{\sum }a_{i}^{}\Delta \theta_{i}^{}e^{-{\left(\Delta \theta_{i}\right)}^{2}/2{\left(b_{i}\right)}^{2}}, \end{array}$$

where *ω* is the angular velocity of the time vector as it moves around the limit circle (representing the period *T*), $\alpha =1-\sqrt{x^{2}+y^{2}}$, *θ* = *a*tan⁡(*y*/*x*), Δ*θ*_*i*_ = *θ* − *θ*_*i*_, *a*_*i*_ contains the amplitudes of the peaks, *b*_*i*_ contains the width of each peak, and *θ*_*i*_ are the angles which specify the *P*-, *Q*-, *R*-, *S*-, *T*-waves/peaks.

Secondly, the PLI components, simulated as sinusoids, are added. Usually the PLI fundamental component is supposed to be constant. However, there are some deviations from the fundamental frequency in real applications, mainly due to unstable power sources. Thus, the PLI components can exhibit significant frequency deviation, up to 3% [30, 97–100] (the deviation differs from country to country, depending on the available power supply technologies).

Three data sets are therefore constructed, considering three PLI scenarios (7): (i) the PLI contains just the fundamental power line interference component, of 50 Hz, (ii) the PLI includes both the power line fundamental frequency and its 3rd harmonic (150 Hz), and (iii) the PLI is a sinusoid whose frequency slightly varies in time around 50 Hz. Consider

(7) PLI(t)={A1·sin(2πf1·t),f1=50 Hz, ideal caseA1·sin(2πf1t)+A3·sin(2πf3·t),f1=50 Hz,  f3=150 Hz,  A3=kA1,A1·sin(2πf1(t)·t),f1(t)=50 Hz±rand(t),  f1(t) exhibits stepwise changes,

where *k* = 20%.

It is clear that the worst scenario is the third one, assuming that the power line fundamental frequency is time-varying.

For each scenario, the SNR defined by (8) is varied; five noise levels are considered: −2 dB, 0 dB, 2 dB, 4 dB, and 5 dB:

(8) $$\begin{array}{c} \text{SNR}=10\;\log_{10}\left(\frac{P_{\text{fECG}}}{P_{\text{PLI}}}\right)=10\;\log_{10}\left(\frac{\sum_{i=1}^{n}\text{fECG}\left(i\right)}{\sum_{i=1}^{n}\text{PLI}\left(i\right)}\right). \end{array}$$

For the qualitative evaluation of the implemented algorithms, the following performance indices are considered as follows.

(a) Normalized root mean square error, expressed in percentage:
  (9) $$\begin{array}{c} \text{RMSD}=\sqrt{\frac{\sum_{i=1}^{N}{\left(\text{orig}\_\text{fECG}(i)-\text{est}\_\text{fECG}(i)\right)}^{2}}{\sum_{i=1}^{N}{\text{orig}\_\text{fECG}(i)}^{2}}}*100. \end{array}$$
(b) Noise retention, expressed in percentage:
  (10) $$\begin{array}{c} \text{NR}=\frac{P_{\text{orig}\_\text{fECG}}-P_{\text{est}\_\text{fECG}}}{P_{\text{orig}\_\text{fECG}}}*100, \end{array}$$
  where *P* is the power of the signal computed with
  (11) $$\begin{array}{c} P_{\text{orig}\_\text{fECG}}=10*\log_{10}\overset{N}{\underset{i=1}{\sum }}{\text{orig}\_\text{fECG}(i)}^{2}. \end{array}$$
(c) SNR improvement [47]:
  (12) SNRimp =10∗log⁡10⁡{∑i=1N[signal(i)−orig_fECG(i)]2∑i=1N[est_fECG(i)−orig_fECG(i)]2},
  where signal is the input signal containing both the fECG and the PLI.
(d) Cross-correlation coefficient, considering the original and the denoised fECG signal:
  (13) $$\begin{array}{c} p=\frac{\sum_{i=1}^{N}\left(\text{est}\_\text{fECG}\left(i\right)*\text{orig}\_\text{fECG}\left(i\right)\right)}{\sqrt{\sum_{i=1}^{N}{\text{est}\_\text{fECG}(i)}^{2}*\sum_{i=1}^{N}{\text{orig}\_\text{fECG}(i)}^{2}}}. \end{array}$$

## 3. Results

**Figure 8:**
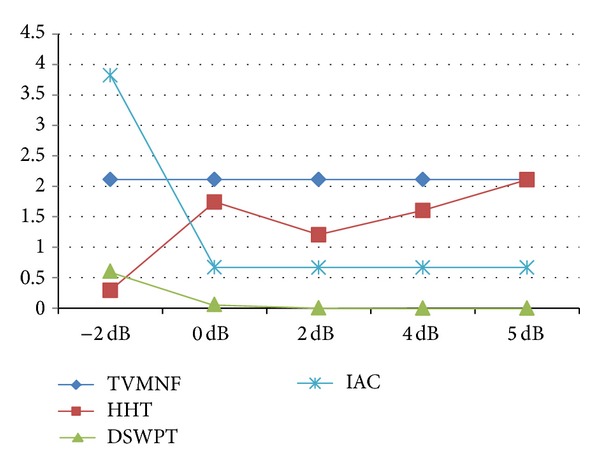
The RMSD (%) for scenario 2 (WNN is excluded).

**Figure 9:**
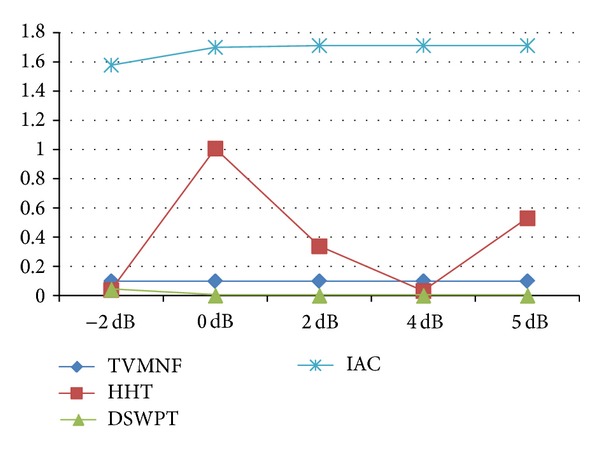
The NR (%) for scenario 2 (WNN is excluded).

**Figure 10:**
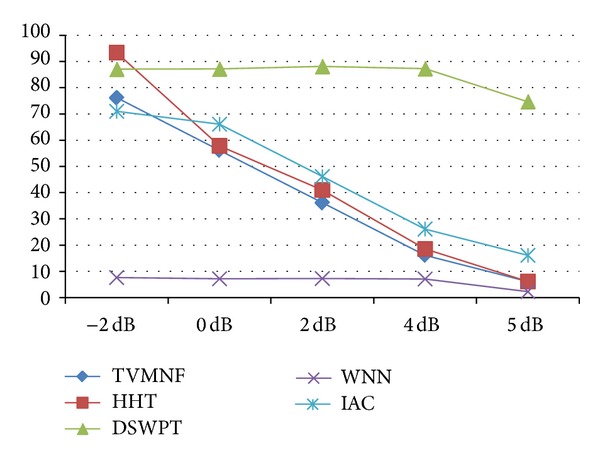
The SRN_imp_ for scenario 2.

**Figure 11:**
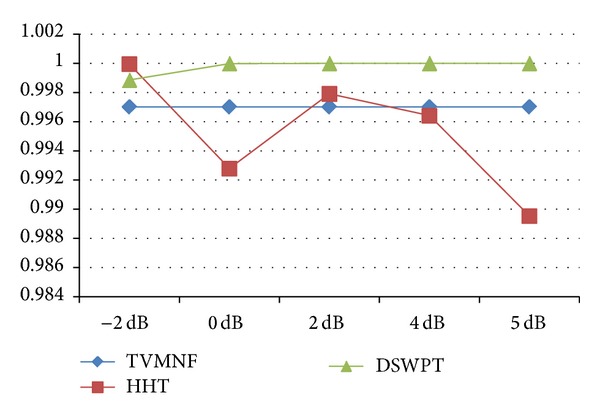
The cross-correlation coefficient for scenario 2 (WNN and IAC are excluded).

The results obtained when applying the five selected algorithms are organized as follows. (i) For each performance index a table is constructed. The columns correspond to the evaluated algorithms and the rows to the scenarios, assuming that the SNR is −2, 0, 2, 4, and 5 dB. The best result obtained for the performance index is emphasized in bold for each case and for each scenario. The scenarios for which some algorithms are not working by principle are represented by empty gray cells. (ii) The performance indices for scenario 2, when the SNR is −2 dB, are illustrated for each algorithm (see Figures 8, 9, 10, and 11). The second scenario is chosen instead of the worst case scenario (scenario 3) because two algorithms are not working by principle in this case. Furthermore, if the algorithms with worst performance have the performance indices very far from the other values (i.e., are outliers), they are excluded from the graphical representation, in order to offer a meaningful comparison of the algorithms. (iii) The results obtained for the selected BSS method (FastICA) are considered apart from the others, because this method has a totally different working principle, assuming that more recorded channels are available. Thus, for each scenario, the FastICA algorithm is evaluated by considering that the available ICA inputs are the five simulated signals, with different SNRs.

## 4. Discussion

In Tables 2, 3, 4, and 5, it can be observed that the worst overall performance is obtained when the WNN is applied. The algorithm is able to reduce the noise if its level is very low, but the QRS complex, containing high frequencies, is disturbed, impairing the fECG morphology analysis. When the signal is hidden by the noise, that is, low level of SNR, the method fails to extract the denoised signal, which can be explained by the thresholding step. The main advantage of the algorithm is the computation time of the testing (denoising) phase, assuming that the neural network is already trained. Because of the overall bad performance the algorithm is excluded from the following discussion.

The TVMNF method proposed by [32] shows good results for scenarios 1 and 2 having a RMSD of 2.11%, Figure 8, and a SNR improvement, SRN_imp_ = 76 dB for the scenario 2 (see Figure 11), SNR = −2 dB (worst scenario). No ringing effect is observed near the QRS complexes. However, despite the good results, the main drawback is that the filter cannot be applied when the fundamental frequency is varying, as expected in real applications. Thus, this type of filter is of limited usage.

As expected, the adaptive methods are able to follow the changes in the frequencies of the PLI components; hence, they can be successfully applied in scenario 3. According to the computed performance indices, the IAC is able to obtain good estimates of the fECG signal when the fundamental frequency is fixed, even when the harmonics are present. However, for the worst SNR, that is, −2 dB, the obtained performance is slightly worse as compared to the performance of the other algorithms (see Figures 8–11). For scenario 3 the algorithm has the worst performance (RMSD = 655% and a noise retention factor of NR = 95%). This is due to the fact that the algorithm needs very long adaptation time (approximately 10 s) when step variations in the PLI fundamental frequency appear. Thus, IAC is of limited use in real applications.

The HHT method implemented in this study shows also good results. However, at small SNR values, small oscillations appear near the QRS complex, suggesting that some very low PLI components can be found in the low order IMFs. Moreover, when the method is applied in scenario 3, outliers appear when the PLI fundamental frequency is changing. The main advantage of this method is that it is suitable for nonstationary signals like the biopotentials and that it is fully data driven; that is, no *a priori* knowledge is necessary. The main disadvantage is that the decomposition does not fully separate the oscillations; thus, some useful information can be found in the IMFs containing the PLI components; in addition, it does not have, by now, a complete mathematical evaluation. However, recent papers present some improvements to the basic method claiming a better decomposition (e.g., Ensemble Empirical Mode Decomposition (EEMD) [101] and Complete Ensemble Empirical Mode Decomposition [102]).

The BSS algorithm is able to separate the fECG from the PLI showing the same performance no matter how the SNR is varying, because by principle it exploits the independence between any two signal sources. However, it should be noted that the comparison with the other method is not quite fair in the current study; in order to have the same simulations, the same signals used for the other algorithms were fed to the FastICA. In real application, the input of ICA algorithm is signals obtained from different channels, meaning different fECG waveforms, which can lower the performance of ICA in fECG extraction. Moreover, the physical relevance of the fECG independent components obtained when applying ICA is still a subject of discussion among researchers. Thus, the method has limited usage in real application, but it can be successfully combined with the adaptive filtering techniques, improving the estimation provided by the PLI reference block.

As theoretically expected, the best performance is obtained when using the DSWPT, if the PLI is stationary and includes exactly the 50 Hz and its harmonics. However, the method does not work in the worst scenario. Since the algorithm estimates the PLI interference assuming that the power line frequency is 50 Hz (in step 2, templates of the sine disturbances are constructed, averaging the segmented signal, using a window of 20 samples), it is expected that the algorithms fail in cancelling the PLI when the frequency is more or less different from 50 Hz, or even worse, when the power line frequency is varying, which is supported by the obtained results.

## 5. Conclusions

In this paper a review of PLI cancelling methods applied in fECG signal processing is proposed, revealing the main concepts provided in the literature for suppressing the 50 Hz/60 Hz component and its harmonics from biosignals. The selected algorithms are quantitatively analyzed, using different performance criteria and practical considerations are provided when discussing the PLI cancelling from abdominal fECGs. Three sets of simulated data are constructed and used in the quantitative evaluation of the algorithms, considering the 50 Hz PLI fundamental frequency, the 50 Hz combined with the 150 Hz PLI component, and a varying PLI fundamental frequency. The quantitative performance is monitored using five different indices, corresponding to different SNRs.

While some methods, like WNN, show very bad performances, most of the algorithms have good results, especially in scenarios 1 and 2. The DSWPT has the best performance in scenarios 1 and 2, as depicted in the Figures 8–11, but its main drawback is that it is not suitable for the most realistic scenario, scenario 3. The HHT based algorithm shows the best performance overall, considering the implemented scenarios. Thus, further studies should concentrate on exploiting the capabilities of the HHT method.

## Figures and Tables

**Table 1:** Main characteristics and findings for the selected algorithms, representative for the PLI cancelling approaches available in the literature.

Authors/year	Title	Type of publication	Category/acronym of the algorithm	Main results
Piskorowski/2012 [32]	Suppressing harmonic powerline interference using multiple-notch filtering methods with improved transient behavior	Review article	Digital fixed notch filters/TVMNF	A new class of digital parameter varying IIR multiple-notch filters with reduced transient response is introduced. The simulations and the quantitative evaluation on suppressing the 60 Hz PLI show that the concept of the filter with time-varying pole radius may be used to improve the dynamic behavior of multiple-notch filter.

Martens et al./2006 [39]	An improved adaptive power line interference canceller for electrocardiography	Research article	Adaptive filters/IAC	An improved version of the adaptive canceller for the reduction of the PLI components is proposed. The algorithm is able to track the frequencies, the amplitudes, and the phases for PLI deviations up to about 4 Hz; it is also insensitive to baseline wander.

Hyvärinen et al./2001 [65]	Independent component analysis: algorithms and applications	Research article	Blind source separation/FastICA	A new ICA algorithm, based on a fixed-point iteration scheme when finding a maximum of the nongaussianity, is proposed. It has some advantages as compared to other existing ICA methods: it finds directly independent components (ICs), which can be estimated one by one; the algorithm is, in addition, parallelizable, computationally simple, and requires little memory space.

Pal and Mitra/2012 [66]	Empirical mode decomposition based ECG enhancement and QRS detection	Research article	EMD and HHT/HHT	A new algorithm is proposed based on EMD methods. The improved algorithm proposed in this article differs from the similar algorithms in the following: the baseline wander is corrected by selective reconstruction from IMFs, considering the slope minimization technique; the noise is removed by eliminating a noisy set of lower order IMFs, based on their power. A statistical peak correction is also performed since the noise cancellation affects the sharp morphology of the peaks. The results reveal that the proposed algorithm shows good detection sensitivity and specificity.

Poungponsri and Yu/2013 [51]	An adaptive filtering approach for electrocardiogram (ECG) signal noise reduction using neural networks	Research article	Neural networks/WNN	In this article the authors propose an adaptive filtering approach based on discrete wavelet transform and artificial neural network. The obtained results, considering simulated data, show that the algorithm can successfully remove various noise and artifacts, leading to a significant SNR improvement, as compared to other algorithms.

Galiana-Merino et al. /2013 [43]	Power line interference filtering on surface electromyography based on the stationary wavelet packet transform	Research article	Wavelet transform/DSWPT	A new method is proposed to estimate and remove PLI components from EMG signals. The method is based on the stationary wavelet packet transform. The quantitative evaluation is performed using synthetic signals, with different SNR values, and the results are compared with the ones obtained when using an adaptive Laguerre filter and other digital filters. In all cases, the proposed algorithm shows an excellent performance, independent of the SNR value.

**Table 2:** The RMSD (%) obtained for each algorithm and for each data set.

Data set	SNR (dB)	Algorithm					
		TVMNF	HHT	DSWPT	WNN	IAC	FastICA
1	−2	2.003515	0.292652	**0.195444**	77.31631	1.77611	0,073811
	0	2.003515	1.740565	**0.018847**	70.02215	0.667624	
	2	2.003515	1.197345	**0.001283**	33.19379	0.666902	
	4	2.003515	1.624253	**0.000778**	5.631514	0.666882	
	5	2.003515	2.09266	**0.000883**	3.244183	0.666879	

2	−2	2.112907	0.292852	**0.60503**	72.03313	3.822223	0,001657
	0	2.112907	1.743529	**0.059846**	68.07016	0.669611	
	2	2.112907	1.205285	**0.005355**	33.1956	0.666898	
	4	2.112907	1.603147	**0.000589**	5.626975	0.666882	
	5	2.112907	2.109169	**0.00079**	3.230891	0.666879	

3	−2		38.81253		64.46775	655.1273	1,269908
	0		1.884043		49.6627	396.5219	
	2		1.185072		47.70798	394.4479	
	4		1.62743		38.65239	393.3756	
	5		1.221664		43.60649	393.305	

**Table 3:** The NR (%) obtained for each algorithm and for each data set.

Data set	SNR (dB)	Algorithm					
		TVMNF	HHT	DSWPT	WNN	IAC	FastICA
1	−2	0.03426	0.038749	**0.023308**	6.295244	1.628744	0,010982
	0	0.03426	1.022943	**0.000225**	4.176215	1.705774	
	2	0.03426	0.333364	6.54***E***− 06	10.28646	1.711423	
	4	0.03426	0.033125	8.93***E***− 06	3.873963	1.711467	
	5	0.03426	0.435546	8.96***E***− 06	2.62773	1.711466	

2	−2	0.098402	0.038435	**0.040232**	4.781289	1.577947	0,000112
	0	0.098402	1.006355	**0.000395**	3.559141	1.699513	
	2	0.098402	0.338354	4.81***E***− 06	10.28173	1.71136	
	4	0.098402	0.032068	8.91***E ***− 06	3.874016	1.711466	
	5	0.098402	0.529579	8.95***E***− 06	2.628339	1.711466	

3	−2		38.94626		3.701535	95.16797	0,024024
	0		0.926928		0.853499	42.01306	
	2		0.320873		1.394364	38.06225	
	4		0.032029		7.147367	37.07462	
	5		0.228292		5.364868	37.0534	

**Table 4:** The SRN_imp_ obtained for each algorithm and for each data set.

Data set	SNR (dB)	Algorithm					
		TVMNF	HHT	DSWPT	WNN	IAC	FastICA
1	−2	66.88941	83.59046	**87.09688**	0.995959	67.90879	89,90204
	0	46.88941	48.10367	**87.41253**	0.457028	56.40755	
	2	26.88941	31.35309	**90.75301**	−0.97771	36.41695	
	4	6.889411	8.704401	**75.09279**	−2.46131	16.41721	
	5	−3.11059	−3.4965	**63.99507**	−7.4033	6.417252	

2	−2	76.16926	93.32959	**87.0233**	7.64809	70.99347	132,618
	0	56.16926	57.83398	**87.11819**	7.199378	66.12334	
	2	36.16926	41.04077	**88.08339**	7.304312	46.15861	
	4	16.16926	18.5631	**87.25321**	7.064304	26.15881	
	5	6.169261	6.180338	**74.70329**	2.304354	16.15886	

3	−2		41.20189		2.384226	16.90442	65,2529
	0		47.44316		3.811226	4.910191	
	2		31.48669		5.564842	0.575438	
	4		8.713179		7.955288	0.242613	
	5		1.236451		7.002494	0.220137	

**Table 5:** Cross-correlation coefficient, *p*, obtained for each algorithm and for each data set.

Data set	SNR (dB)	Algorithm					
		TVMNF	HHT	DSWPT	WNN	IAC	FastICA
1	−2	0.997565	0.999947	**0.999343**	0.000708	0.607869	0,999997
	0	0.997565	0.994679	**0.999996**	0.002489	0.992365	
	2	0.997565	0.99794	**1**	0.348027	0.987873	
	4	0.997565	0.996368	**1**	0.976249	0.999875	
	5	0.997565	0.990019	**1**	0.987159	0.999987	

2	−2	0.997018	**0.999947**	0.998855	0.000667	0.464252	1
	0	0.997018	0.992789	**0.999991**	0.002342	0.984898	
	2	0.997018	0.997911	**1**	0.347902	0.999995	
	4	0.997018	0.996415	**1**	0.976263	0.999999	
	5	0.997018	0.989539	**1**	0.987179	1	

3	−2		0.313497		−0.00072	0.00938	0,999191
	0		0.992068		−0.00131	0.073122	
	2		0.997936		0.005998	0.187229	
	4		0.996377		0.004007	0.205138	
	5		0.997482		0.010274	0.237579	

## References

[1] Woods JR Case #680 Topic: Fetal Heart Rate Monitoring. http://www.perifacts.eu/cases/Case_680_Fetal_Heart_Rate_Interpretation.php.

[2] Hinshaw K, Ullal A (2007). Peripartum and intrapartum assessment of the fetus. *Anaesthesia and Intensive Care Medicine*.

[3] Ross MG, Devoe LD, Rosen KG (2004). ST-segment analysis of the fetal electrocardiogram improve fetal heart rate tracing interpretation and clinical decision making. *Journal of Maternal-Fetal and Neonatal Medicine*.

[4] Westgate J, Harris M, Curnow JSH, Greene KR (1993). Plymouth randomized trial of cardiotocogram only versus ST waveform plus cardiotocogram for intrapartum monitoring in 2400 cases. *American Journal of Obstetrics and Gynecology*.

[5] Amer-Wåhlin I, Hellsten C, Norén H (2001). Cardiotocography only versus cardiotocography plus ST analysis of fetal electrocardiogram for intrapartum fetal monitoring: A Swedish randomised controlled trial. *Lancet*.

[6] Ojala K, Vääräsmäki M, Mäkikallio K, Valkama M, Tekay A (2006). A comparison of intrapartum automated fetal electrocardiography and conventional cardiotocography—A Randomised Controlled Study. *International Journal of Obstetrics and Gynaecology*.

[7] Vayssiere C, David E, Meyer N (2007). A French randomized controlled trial of ST segment analysis in a population with abnormal cardiotocograms during labor. *American Journal of Obstetrics & Gynecology*.

[8] Westerhuis ME, Visser GH, Moons KG (2010). Cardiotocography plus ST analysis of fetal electrocardiogram compared with cardiotocography only for intrapartum monitoring: a randomized controlled trial. *Obstetrics & Gynecology*.

[9] Norén H, Blad S, Carlsson A (2006). STAN in clinical practice-The outcome of 2 years of regular use in the city of Gothenburg. *American Journal of Obstetrics and Gynecology*.

[10] Norén H, Luttkus AK, Stupin JH (2007). Fetal scalp pH and ST analysis of the fetal ECG as an adjunct to cardiotocography to predict fetal acidosis in labor: A multi-center, case controlled study. *Journal of Perinatal Medicine*.

[11] Massoud M, Giannesi A, Amabile N, Manevy M, Geron G, Gaucherand P (2007). Fetal electrocardiotocography in labor and neonatal outcome: An observational study in 1889 patients in the French center of Edouard Herriot, Lyon. *Journal of Maternal-Fetal and Neonatal Medicine*.

[12] Palmgren Colov NS (2007). Need for extensive education when implementing new foetal monitoring technology. *Ugeskrift for Laeger*.

[13] Melin M, Bonnevier A, Cardell M, Hogan L, Herbst A (2008). Changes in the ST-interval segment of the fetal electrocardiogram in relation to acid-base status at birth. *International Journal of Obstetrics and Gynaecology*.

[14] Welin A-K, Norán H, Odeback A, Andersson M, Andersson G, Rosén KG (2007). STAN, a clinical audit: The outcome of 2 years of regular use in the city of Varberg, Sweden. *Acta Obstetricia et Gynecologica Scandinavica*.

[15] Kale A, Chong Y-S, Biswas A (2008). Effect of availability of fetal ECG monitoring on operative deliveries. *Acta Obstetricia et Gynecologica Scandinavica*.

[16] Norén H, Carlsson A (2010). Reduced prevalence of metabolic acidosis at birth: an analysis of established STAN usage in the total population of deliveries in a Swedish district hospital. *American Journal of Obstetrics and Gynecology*.

[17] Rzepka R, Torbé A, Kwiatkowski S, Blogowski W, Czajka R (2010). Clinical outcomes of high-risk labours monitored using fetal electrocardiography. *Annals of the Academy of Medicine Singapore*.

[18] Ragupathy K, Ismail F, Nicoll AE (2010). The use of STAN monitoring in the labour ward. *Journal of Obstetrics and Gynaecology*.

[19] Signorini MG, Magenes G, Cerutti S, Arduini D (2003). Linear and nonlinear parameters for the analysis of fetal heart rate signal from cardiotocographic recordings. *IEEE Transactions on Biomedical Engineering*.

[20] Neilson JP (2012). Fetal electrocardiogram (ECG) for fetal monitoring during labour. *Cochrane Database of Systematic Reviews*.

[21] Cohen WR, Ommani S, Hassan S (2012). Accuracy and reliability of fetal heart rate monitoring using maternal abdominal surface electrodes. *Acta Obstetricia et Gynecologica Scandinavica*.

[22] Jezewski J, Wrobel J, Horoba K (2006). Comparison of Doppler ultrasound and direct electrocardiography acquisition techniques for quantification of fetal heart rate variability. *IEEE Transactions on Biomedical Engineering*.

[23] Hasan MA, Reaz MBI, Ibrahimy MI, Hussain MS, Uddin J (2009). Detection and processing techniques of FECG signal for fetal monitoring. *Biological Procedures Online*.

[24] Peters M, Crowe J, Piéri J-F (2001). Monitoring the fetal heart non-invasively: a review of methods. *Journal of Perinatal Medicine*.

[25] Huhta JC, Webster JG (1973). 60-Hz interference in electrocardiography. *IEEE Transactions on Biomedical Engineering*.

[26] Villablanca ME (2009). Harmonic-free line-commutated ac/dc rectifiers. *Electric Power Systems Research*.

[27] Jain SK, Singh SN (2011). Harmonics estimation in emerging power system: key issues and challenges. *Electric Power Systems Research*.

[28] Wada K, Shimizu T Mitigation method of 3rd-harmonic voltage for a three-phase four-wire distribution system based on a series active filter for the neutral conductor.

[29] Metting van Rijn AC, Peper A, Grimbergen CA (1990). High-quality recording of bioelectric events. Part 1. Interference reduction, theory and practice. *Medical and Biological Engineering and Computing*.

[30] Levkov C, Mihov G, Ivanov R, Daskalov I, Christov I, Dotsinsky I (2005). Removal of power-line interference from the ECG: a review of the subtraction procedure. *BioMedical Engineering Online*.

[31] Kligfield P, Gettes LS, Bailey JJ (2007). Recommendations for the Standardization and Interpretation of the Electrocardiogram: Part I: The Electrocardiogram and Its Technology A Scientific Statement From the American Heart Association Electrocardiography and Arrhythmias Committee, Council on Clinical Cardiology; the American College of Cardiology Foundation; and the Heart Rhythm Society Endorsed by the International Society for Computerized Electrocardiology. *Journal of the American College of Cardiology*.

[32] Piskorowski J (2012). Suppressing harmonic powerline interference using multiple-notch filtering methods with improved transient behavior. *Measurement*.

[33] Joshi YV, Dutta Roy SC (1998). Design of IIR multiple notch filters. *International Journal of Circuit Theory and Applications*.

[34] Deshpande R, Kumar B, Jain SB (2012). On the design of multi notch filters. *International Journal of Circuit Theory and Applications*.

[35] Deshpande R, Kumar B, Jain SB (2010). Highly narrow rejection bandwidth finite impulse response notch filters for communication. *IET Communications*.

[36] Piskorowski J (2010). Digital Q-varying notch IIR filter with transient suppression. *IEEE Transactions on Instrumentation and Measurement*.

[37] Badreldin IS, El-Kholy DS, El-Wakil AA A modified adaptive noise canceler for electrocardiography with no power-line reference.

[38] Badreldin IS, El-Kholy DS, Elwakil AA Harmonic adaptive noise canceler for electrocardiography with no power-line reference.

[39] Martens SMM, Mischi M, Oei SG, Bergmans JWM (2006). An improved adaptive power line interference canceller for electrocardiography. *IEEE Transactions on Biomedical Engineering*.

[40] Maniruzzaman M, Billah KMS, Biswas U, Gain B Least-Mean-Square algorithm based adaptive filters for removing power line interference from ECG signal.

[41] Widrow B, Glover JR, McCool JM (1975). Adaptive noise cancelling: principles and applications. *Proceedings of the IEEE*.

[42] Liangling G, Nanquan Z, Haotian W (2011). Application of interference canceller in bioelectricity signal disposing. *Procedia Environmental Sciences*.

[43] Galiana-Merino JJ, Ruiz-Fernandez D, Martinez-Espla JJ (2013). Power line interference filtering on surface electromyography based on the stationary wavelet packet transform. *Computer Methods and Programs in Biomedicine*.

[44] Karthikeyan P, Murugappan M, Yaacob S (2012). ECG signal denoising using wavelet thresholding techniques in Human Stress Assessment. *International Journal on Electrical Engineering and Informatics*.

[45] Alfaouri M, Daqrouq K (2008). ECG signal denoising by wavelet transform thresholding. *American Journal of Applied Sciences*.

[46] Pal S, Mitra M (2012). Empirical mode decomposition based ECG enhancement and QRS detection. *Computers in Biology and Medicine*.

[47] Kabir MA, Shahnaz C (2012). Denoising of ECG signals based on noise reduction algorithms in EMD and wavelet domains. *Biomedical Signal Processing and Control*.

[48] Artūras J, Vaidotas M, Arūnas L (2012). Ensemble empirical mode decomposition based feature enhancement of cardio signals. *Medical Engineering & Physics*.

[49] Li G, Zeng X, Zhou X, Zhou Y, Liu G, Zhou X (2012). Robust suppression of nonstationary power-line interference in electrocardiogram signals. *Physiological Measurement*.

[50] Hajimolahoseini H, Taban MR, Soltanian-Zadeh H (2012). Extended Kalman Filter frequency tracker for nonstationary harmonic signals. *Measurement*.

[51] Poungponsri S, Yu X-H (2013). An adaptive filtering approach for electrocardiogram (ECG) signal noise reduction using neural networks. *Neurocomputing*.

[52] Mateo J, Sánchez C, Torres A, Cervigon R, Rieta JJ Neural network based canceller for powerline interference in ECG signals.

[53] Lin Y-D, Hsu C-Y, Chen H-Y, Tseng K-K (2013). Efficient block-wise independent component analysis with optimal learning rate. *Neurocomputing*.

[54] Chawla MPS (2011). PCA and ICA processing methods for removal of artifacts and noise in electrocardiograms: a survey and comparison. *Applied Soft Computing Journal*.

[55] Hyvärinen A (1999). Fast and robust fixed-point algorithms for independent component analysis. *IEEE Transactions on Neural Networks*.

[56] Allen DP (2009). A frequency domain Hampel filter for blind rejection of sinusoidal interference from electromyograms. *Journal of Neuroscience Methods*.

[57] Levkov C, Mihov G, Ivanov R, Daskalov I, Christov I, Dotsinsky I (2005). Removal of power-line interference from the ECG: a review of the subtraction procedure. *BioMedical Engineering Online*.

[58] Mihov G, Dotsinsky I, Georgieva T (2005). Subtraction procedure for powerline interference removing from ECG: improvement for non-multiple sampling. *Journal of Medical Engineering and Technology*.

[59] Joshi YV, Dutta Roy SC (1998). Design of IIR multiple notch filters. *International Journal of Circuit Theory and Applications*.

[60] Hamilton PS (1996). A comparison of adaptive and nonadaptive filters for reduction of power line interference in the ECG. *IEEE Transactions on Biomedical Engineering*.

[61] Luo S, Johnston P (2010). A review of electrocardiogram filtering. *Journal of Electrocardiology*.

[62] American National Standard ANSI/AAMI EC11 (2007). *Diagnostic Electrocardiographic Devices*.

[63] International Standard IEC 60601-1 (2005). *Medical Electrical Equipment–Part 1: General Requirements for Basic Safety and Essential Performance*.

[64] Pei S-C, Tseng C-C (1995). Elimination of AC interference in electrocardiogram using IIR notch filter with transient suppression. *IEEE Transactions on Biomedical Engineering*.

[65] Hyvärinen A, Karhunen J, Oja E (2001). *Independent Component Analysis*.

[66] Pal S, Mitra M (2012). Empirical mode decomposition based ECG enhancement and QRS detection. *Computers in Biology and Medicine*.

[67] Wan H, Fu R, Shi L (2006). The Elimination of 50 Hz power line interference from ECG using a variable step size LMS adaptive filtering algorithm. *Life Science Journal*.

[68] Costa MH, Tavares MC (2009). Removing harmonic power line interference from biopotential signals in low cost acquisition systems. *Computers in Biology and Medicine*.

[69] Ziarani AK, Konrad A (2002). A nonlinear adaptive method of elimination of power line interference in ECG signals. *IEEE Transactions on Biomedical Engineering*.

[70] Hyvärinen A, Oja E (1997). A fast fixed-point algorithm for independent component analysis. *Neural Computation*.

[71] Cardoso J-F (1997). Infomax and maximum likelihood for blind source separation. *IEEE Signal Processing Letters*.

[72] Pearlmutter BA, Parra LC (1997). Maximum likelihood blind source separation: a context-sensitive generalization of ICA. *Advances in Neural Information Processing Systems*.

[73] De Lathauwer L, De Moor B, Vandewalle J (2000). Fetal electrocardiogram extraction by blind source subspace separation. *IEEE Transactions on Biomedical Engineering*.

[74] Zarzoso V, Nandi AK (2001). Noninvasive fetal electrocardiogram extraction: blind separation versus adaptive noise cancellation. *IEEE Transactions on Biomedical Engineering*.

[75] Vrins F, Jutten C, Verleysen M (2004). Sensor array and electrode selection for non-invasive fetal electrocardiogram extraction by independent component analysis. *Independent Component Analysis and Blind Signal Separation*.

[76] Sameni R (July 2008). *Extraction of fetal cardiac signals from an array of maternal abdominal recordings [Ph.D. dissertation]*.

[77] Sameni R, Clifford GD (2010). A review of fetal ECG signal processing, No.s and promising directions. *The Open Pacing, Electrophysiology & Therapy Journal*.

[78] Camargo-Olivares JL, Martín-Clemente R, Hornillo-Mellado S, Elena MM, Román I (2011). The maternal abdominal ECG as input to MICA in the fetal ECG extraction problem. *IEEE Signal Processing Letters*.

[79] Cardoso J-F Multidimensional independent component analysis.

[80] Huang NE, Shen Z, Long SR (1998). The empirical mode decomposition and the Hubert spectrum for nonlinear and non-stationary time series analysis. *Proceedings of the Royal Society A*.

[81] Huang NE, Wu ML, Long SR (2003). A confidence limit for the empirical mode decomposition and the Hilbertspectral analysis. *Proceedings of the Royal Society A*.

[82] Blanco-Velasco M, Weng B, Barner KE (2008). ECG signal denoising and baseline wander correction based on the empirical mode decomposition. *Computers in Biology and Medicine*.

[83] Zhang C, Li X, Zhang M A novel ECG signal denoising method based on Hilbert-Huang Transform.

[84] Jiang W, Kong SG, Peterson GD ECG signal classification using block-based neural networks.

[85] Jezewski M, Wrobel J, Labaj P Some practical remarks on neural networks approach to fetal cardiotocograms classification.

[86] Hassan MA, Ibrahimy MI, Reaz MBI (2009). An efficient method for fetal electrocardiogram extraction from the abdominal electrocardiogram signal. *Journal of Computer Science*.

[87] Dey N, Dash TP, Dash S (2011). ECG signal denoising by Functional Link Artificial Neural Network (FLANN). *International Journal of Biomedical Engineering and Technology*.

[88] Zhang Q, Benveniste A (1992). Wavelet networks. *IEEE Transactions on Neural Networks*.

[89] Poungponsri S, Yu X-H Electrocardiogram (ECG) signal modeling and noise reduction using wavelet neural networks.

[90] Moody GB, Mark RG (2001). The impact of the MIT-BIH arrhythmia database. *IEEE Engineering in Medicine and Biology Magazine*.

[91] El-Dahshan E-SA (2011). Genetic algorithm and wavelet hybrid scheme for ECG signal denoising. *Telecommunication Systems*.

[92] Kezi Selva Vijila C, Ebbie Selva Kumar C (2009). Interference cancellation in EMG signal Using ANFIS. *International Journal of Recent Trends in Engineering*.

[93] Karthikeyan P, Murugappan M, Yaacob S (2012). ECG signal denoising using wavelet thresholding techniques in human stress assessment. *International Journal on Electrical Engineering and Informatics*.

[94] Garg G, Gupta S, Singh V, Gupta JRP, Mittal AP Identification of optimal wavelet-based algorithm for removal of power line interferences in ECG signals.

[95] McSharry PE, Clifford GD, Tarassenko L, Smith LA (2003). A dynamical model for generating synthetic electrocardiogram signals. *IEEE Transactions on Biomedical Engineering*.

[96] McSharry PE, Clifford GD ECGSYN—a realistic ECG waveform generator. http://www.physionet.org/physiotools/ecgsyn/.

[97] Chang K-M (2010). Arrhythmia ECG noise reduction by ensemble empirical mode decomposition. *Sensors*.

[98] Tabakov S, Iliev I, Krasteva V (2008). Online digital filter and QRS detector applicable in low resource ECG monitoring systems. *Annals of Biomedical Engineering*.

[99] McManus CD, Neubert K-D, Cramer E (1993). Characterization and elimination of AC noise in electrocardiograms: a comparison of digital filtering methods. *Computers and Biomedical Research*.

[100] Kumaravel N, Senthil A, Sridhar KS, Nithiyanandam N (1995). Integrating the ECG power-line interference removal methods with rule-based system. *Biomedical Sciences Instrumentation*.

[101] Wu Z, Huang NE (2009). Ensemble empirical mode decomposition: a noise-assisted data analysis method. *Advances in Adaptive Data Analysis*.

[102] Lin J (2012). Improved ensemble empirical mode decomposition and its applications to gearbox fault signal processing. *International Journal of Computer Science*.

